# The Agreement between Feline Pancreatic Lipase Immunoreactivity and DGGR-Lipase Assay in Cats—Preliminary Results

**DOI:** 10.3390/ani11113172

**Published:** 2021-11-06

**Authors:** Magdalena Maria Krasztel, Michał Czopowicz, Olga Szaluś-Jordanow, Agata Moroz, Marcin Mickiewicz, Jarosław Kaba

**Affiliations:** 1 Division of Veterinary Epidemiology and Economics, Institute of Veterinary Medicine, Warsaw University of Life Sciences-SGGW, Nowoursynowska 159c, 02-776 Warsaw, Poland; magda.krasztel@gmail.com (M.M.K.); agata_moroz@sggw.edu.pl (A.M.); marcin_mickiewicz@sggw.edu.pl (M.M.); jaroslaw_kaba@sggw.edu.pl (J.K.); 2 Department of Small Animal Diseases with Clinic, Institute of Veterinary Medicine, Warsaw University of Life Sciences-SGGW, Nowoursynowska 159c, 02-776 Warsaw, Poland; olga_szalus_jordanow@sggw.edu.pl

**Keywords:** AC_1_ coefficient, beyond chance agreement, chance-corrected agreement, Cohen’s kappa coefficient, fPLI, pancreatitis

## Abstract

**Simple Summary:**

Feline pancreatitis is a common disease with diagnostics requiring a combination of clinical, laboratory and imaging methods. Measuring of pancreatic lipase concentration is the laboratory mainstay of feline pancreatitis diagnostics. It can be done using immunoenzymatic assay (feline pancreatic immunoreactivity, fPLI) or colorimetric assay based on hydrolyzing a special substrate (DGGR) to a color product. A DGGR-lipase assay is manufactured by various companies. Studies carried out in cats on the DGGR-lipase assay of a single manufacturer have shown a high agreement with fPLI, however studies in dogs indicate that the performance of assays of different manufacturers may vary considerably. One of the largest Polish veterinary laboratories working 24/7 in the largest Polish cities offers the DGGR-lipase assay of a large Polish laboratory solutions provider. The values obtained in this assay are higher than those yielded by the traditional DGGR-lipase assay validated in studies carried out so far. Therefore, we decided to evaluate the agreement between the DGGR-lipase assay of the Polish manufacturer and fPLI. The agreement proved to be as high as for the traditional assay.

**Abstract:**

The colorimetric catalytic assay based on the use of 1,2-o-dilauryl-rac-glycero-3-glutaric acid-(6′-methylresorufin) (DGGR) ester as a substrate for pancreatic lipase activity is commonly used for the diagnosis of pancreatitis in dogs and cats. Even though the assay has generally been shown to yield consistent results with feline pancreatic lipase immunoreactivity (fPLI) assay, the agreement may vary between assays of different manufacturers. In this study, the chance-corrected agreement between a DGGR-lipase assay offered by one of the biggest providers of diagnostic solutions in Poland and fPLI assay was investigated. The study was carried out on 50 cats in which DGGR-lipase activity and fPLI were tested in the same blood sample. The chance-corrected agreement was determined using Gwet’s AC_1_ coefficient separately for the fPLI assay’s cut-off values of >3.5 μg/L and >5.3 μg/L. The DGGR-lipase activity significantly positively correlated with fPLI (R_s_ = 0.665; CI 95%: 0.451, 0.807, *p* < 0.001). The chance-corrected agreement between the fPLI assay and DGGR-lipase assay differed considerably depending on the cut-off values of the DGGR-lipase assay. When the cut-off value reported in the literature (>26 U/L) was used, it was poor to fair. It was moderate at the cut-off value recommended by the laboratory (>45 U/L), and good at the cut-off value recommended by the assay’s manufacturer (>60 U/L). The highest agreement was obtained between the fPLI assay at the cut-off value of 3.5 μg/L and the DGGR-lipase assay at the cut-off value of 55 U/L (AC_1_ = 0.725; CI 95%: 0.537, 0.914) and between the fPLI assay at the cut-off value of 5.3 μg/L and the DGGR-lipase assay at the cut-off value of 70 U/L (AC_1_ = 0.749; CI 95%: 0.577, 0.921). The study confirms that the chance-corrected agreement between the two assays is good. Prospective studies comparing both assays to a diagnostic gold standard are needed to determine which of them is more accurate.

## 1. Introduction

Diagnosing feline pancreatitis is challenging as the tests used in clinical practice have limited accuracy when compared to histopathology, which is considered to be the gold standard [1]. At present, diagnosis is made in daily medicine based on clinical signs, pancreatic lipase concentration or activity and the appearance of the pancreas and surrounding mesentery in the abdominal ultrasound examination [1]. As clinical signs are very unspecific with predominating lethargy and anorexia, followed by vomiting and abdominal pain [1,2,3], the combination of ultrasound and laboratory tests plays a crucial role [1]. Pancreatic lipase concentration is quantified using ELISA as feline pancreatic lipase immunoreactivity (fPLI). The assay was initially developed in the Gastrointestinal Laboratory at Texas A&M University as a radioimmunoassay based on polyclonal antibodies, and in 2008 was it replaced by a commercial ELISA based on monoclonal antibodies (Spec fPL^TM^; Westbrook, ME, USA) [4] offered by the IDEXX Laboratories (Westbrook, ME, USA). Recently, an fPLI assay of Laboklin (Bad Kissingen, Germany) has been introduced onto the market [5]. fPLI results are currently interpreted according to the reference interval (RI) of ≤3.5 μg/L and the cut-off value indicating pancreatitis of >5.3 μg/L; however, these are based on a small-scale retrospective study available only in the form of an abstract and never published as a peer-reviewed article [6]. As the fPLI assay is currently only offered by two commercial laboratories, it is relatively expensive and has a turnaround time of 1–3 days. An in-clinic point-of-care fPLI test (Snap fPL^TM^; Westbrook, ME, USA), although useful in emergency practice, provides only binary results with the cut-off value set at an fPLI of 3.5 μg/L [7]. 

In 2001, even before the development of the fPLI assay, the colorimetric catalytic assay based on the use of 1,2-o-dilauryl-rac-glycero-3-glutaric acid-(6′-methylresorufin) (DGGR) ester as a substrate for pancreatic lipase activity was developed in response to a very low specificity of available assays for serum lipase [8]. Despite a recent study implying that it lacks analytical specificity for pancreatic lipase [9], several large-scale studies have demonstrated its moderate to high agreement with fPLI assay [10,11,12], moderate diagnostic accuracy compared to histopathological examination, non-inferior to the accuracy of fPLI assay [12] as well as its resistance to the influence of concurrent azotemia [10,13,14]. A DGGR-lipase assay evaluated in these studies was manufactured by Roche Diagnostics (Rotkreuz, Switzerland) and based on these results, as well as thanks to its relatively low price and immediate availability of results [15], this DGGR-lipase assay has been incorporated into a routine feline clinical chemistry panel by most veterinary laboratories worldwide. It is usually interpreted according to the RI of ≤26 U/L, established using 80 apparently healthy cats of various breeds and either sex [10], although this figure should be considered valid only for the DGGR-lipase assay of this particular manufacturer.

A DGGR-lipase assay is offered by virtually all commercial veterinary laboratories in Poland. Most of them use the Roche Diagnostics assay, however some quantify lipase activity using a DGGR-lipase assay manufactured by PZ Cormay S.A. (Łomianki, Poland), a large Polish provider of diagnostic solutions, present on the market of many European and Asian countries. Our clinical observations, based on a long cooperation with the biggest Polish laboratory currently offering this assay in Poland’s seven largest cities, indicate that the lipase activity measurements obtained in this assay are higher than those obtained in the Roche Diagnostics assay; however, they still seem to be consistent with the results of the fPLI assay. Therefore, we decided to evaluate the chance-corrected (beyond chance) agreement between the PZ Cormay S.A. (Łomianki, Poland) DGGR-lipase assay and the IDEXX Laboratories fPLI assay and determine the cut-off values of this DGGR-lipase assay where the agreement was highest.

## 2. Materials and Methods

### 2.1. Study Design and Laboratory Measurements

It was a retrospective study. The data were collected in two private small animal veterinary clinics in central Poland. We searched the databases for the laboratory results of cats presented to clinics in 2018–2019 whose blood samples had been sent to commercial veterinary laboratories for quantification of fPLI, DGGR-lipase activity, as well as a basic clinical chemistry panel. Only results measured from the blood samples that were obtained during the same blood collection and were immediately sent to the laboratories (without freezing) were included in the analysis. The cats presented at the clinics on the account of various health problems as well as for routine health checks. However, information on the clinical characteristics, diagnosis, and medication used was not available in this study.

The serum pancreatic lipase concentration [μg/L] was quantified as fPLI using ELISA based on monoclonal antibodies (Spec fPL™) in IDEXX Laboratories GmbH (Ludwigsburg, Germany). The fPLI reference interval (RI) was ≤3.5 μg/L, and the cut-off value for diagnosing pancreatitis was >5.3 μg/L [6].

The serum lipase activity [U/L] was measured with the DGGR-lipase assay (Prestige 24i LQ Lipase, PZ Cormay S.A., Łomianki, Poland). An analytical sensitivity (limit of detection, LOD) of the assay was 7 U/L, run-to-run repeatability and day-to-day repeatability (precision) expressed as the coefficient of variation (CV%) were from 2.3% to 5.5%, and from 2.1% to 3.4%, respectively, and linearity was ensured up to 600 U/L according to the manufacturer’s information. All biochemical measurements were performed in an automatic photometric clinical chemistry analyzer ACCENT-200 (PZ Cormay S.A., Łomianki, Poland) in the LAB-WET veterinary laboratory in Warsaw, Poland. The RI for DGGR-lipase assay recommended by the laboratory was ≤45 U/L while the RI reported in the assay’s manufacturer’s manual was ≤60 U/L. 

Given the potential influence of kidney function [13,14] and serum lipid concentration [8] on the results of lipase assays (mainly DGGR-lipase assay), the link between creatinine, cholesterol, and triglycerides concentration on one side and the two lipase assays on the other side was measured and the cats were divided into subpopulations based on the concentration of creatinine (non-azotemic when ≤1.8 mg/dL, otherwise azotemic), total cholesterol (normocholesterolemic when ≤200 mg/dL, otherwise hypercholesterolemic), and triglycerides (normotriglyceridemic when ≤160 mg/dL, otherwise hypertriglyceridemic). Laboratory RIs were used [16].

### 2.2. Statistical Analysis

Numerical variables were expressed as the median, interquartile range (IQR) and range. As laboratory measurements were right-hand skewed, correlation was determined using the Spearman’s rank correlation coefficient (R_s_) and the 95% confidence interval (CI 95%) was calculated according to Bonnett and Wright method [17]. A significance level (α) was set at 0.05. Statistical analyses were performed in TIBCO Statistica 13.3 (TIBCO Software Inc., Palo Alto, CA, USA). 

The chance-corrected agreement was determined using Gwet’s AC_1_ coefficient [18,19], a paradox-resistant alternative to Cohen’s kappa (κ) coefficient [20], according to the commonly accepted formula:

AC_1_ = (P_o_ − P_e_(γ))/(1 − P_e_(γ)) = 1 − [(1 − P_o_)/(1 − P_e_(γ))] (1)

where P_o_ was an observed agreement calculated as the sum of probabilities of consistent positive results (p_++_) and consistent negative results (p_--_) of both tests:

P_o_ = p_++_ + p_--_ (2)

and P_e_(γ) was an expected chance agreement calculated according to the formula proposed by Gwet [18,19]:

P_e_(γ) = 0.5 × (p_+._ + p_.+_) × [2 − (p_+._ + p_.+_)] (3)

where p_+_ signified the probability of a positive result of test 1 (T1) and p_+_ signified the probability of a positive result of test 2 (T2). Step-by-step explanation of the calculations is given in Table S2. 

To select the cut-off value of DGGR-lipase assay at which the chance-corrected agreement with fPLI assay was the highest, AC_1_ coefficient was calculated for a cut-off value set at each observed result of the DGGR-lipase assay and the cut-off value associated with the highest AC_1_ coefficient value was considered optimal.

Chance-corrected agreement was categorized as follows: AC_1_ = 0.81–1.0–very good agreement, 0.61–0.80–good (substantial), 0.41–0.60–moderate, 0.21–0.40–fair, ≤0.20–poor [21]. To enable comparison of the results with other studies, the Cohen’s κ is provided in Table S3.

## 3. Results

The study population included 50 cats, 29 neutered males and 21 spayed females, aged from 1 to 19 years with a median age (IQR) of 12 (10 to 13) years. The breeds included Domestic Shorthair (36 cats), Sphynx (4 cats), Russian, Siberian, Devon Rex, Maine Coon (2 cats each), Ragdoll and British Shorthair (1 cat each).

fPLI ranged from 0.6 to 50 μg/L with a median (IQR) of 3.5 (1.3 to 8.9) μg/L. fPLI was within the RI (≤3.5 μg/L) in 26 cats (52%) and >5.3 μg/L in 20 cats (40%). The DGGR-lipase activity ranged from 17 to 264 U/L with a median (IQR) of 51 (32 to 72) U/L. It was ≤26 U/L in only 7 cats (14%), ≤45 U/L in 21 cats (42%), and ≤60 U/L in 33 cats (66%).

Creatinine concentration ranged from 0.8 to 8.0 mg/dL with a median (IQR) of 1.3 (1.0 to 2.0) mg/dL. Azotemia (creatinine concentration > 1.8 mg/dL) was detected in 15 cats (30%). Cholesterol concentration ranged from 116 to 487 mg/dL with a median (IQR) of 202 (149 to 263) mg/dL. Hypercholesterolemia (cholesterol concentration > 200 mg/dL) was detected in 25 cats (50%). Triglyceride concentration ranged from 17 to 851 mg/dL with a median (IQR) of 75 (49 to 126) mg/dL. Hypertriglyceridemia (triglyceride concentration > 160 mg/dL) was detected in 10 cats (20%).

**Figure 1:**
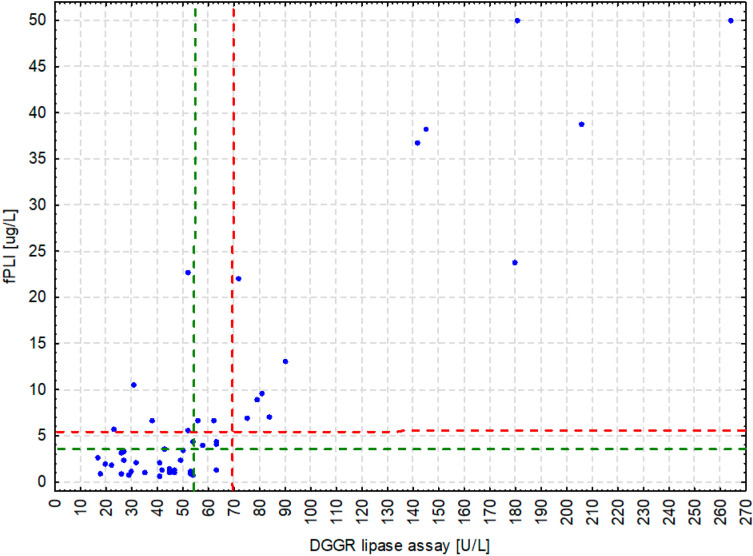
Scatter plot presenting moderate correlation (expressed as the Spearman’s rank correlation coefficient [R_s_]) between feline pancreatic lipase immunoreactivity (fPLI) and DGGR-lipase assay. Broken lines indicate cut-off values: green-3.5 μg/L for fPLI and 55 U/L for the DGGR-lipase assay; red-5.3 μg/L for fPLI and 70 U/L for the DGGR-lipase assay.

The DGGR-lipase activity significantly positively correlated with fPLI (R_s_ = 0.665; CI 95%: 0.451, 0.807, *p* < 0.001; Figure 1). Both fPLI and DGGR-lipase activity significantly positively correlated with creatinine concentration (R_s_ = 0.430; CI 95%: 0.159, 0.640, *p* = 0.002 and R_s_ = 0.452; CI 95%: 0.185, 0.657, *p* = 0.001, respectively) and cholesterol concentration (R_s_ = 0.368; CI 95%: 0.090, 0.592, *p* = 0.009 and R_s_ = 0.341; CI 95%: 0.061, 0.571, *p* = 0.015, respectively). However, they did not correlate with triglyceride concentration (R_s_ = 0.008; CI 95%: −0.271, 0.285, *p* = 0.958 and R_s_ = −0.090; CI 95%: −0.360, 0.194, *p* = 0.534, respectively). 

The chance-corrected agreement between the fPLI assay and DGGR-lipase assay differed considerably depending on the DGGR-lipase assay cut-off values (Table 1). When the cut-off value reported in the literature (>26 U/L) was used, it was poor to fair. It was moderate at the cut-off value recommended by the laboratory (>45 U/L) and good at the cut-off value recommended by the assay’s manufacturer (>60 U/L). The highest agreement was obtained between the fPLI assay at a cut-off value of 3.5 μg/L and the DGGR-lipase assay at a cut-off value of 55 U/L (AC_1_ = 0.73), and between the fPLI assay at a cut-off value of 5.3 μg/L and the DGGR-lipase assay at a cut-off value of 70 U/L (AC_1_ = 0.75). Detailed contingency tables and calculations are presented in Table S3.

At the optimal DGGR-lipase assay cut-off values, the chance-corrected agreement remained good in azotemic and non-azotemic cats, hyper- and normocholesterolemic cats, as well as hyper- and normotriglyceridemic cats (Table 2).

## 4. Discussion

Our study shows that the chance-corrected agreement between the DGGR-lipase assay manufactured by PZ Cormay S.A. (Łomianki, Poland) and the fPLI assay is moderate at the cut-off value used by the laboratory (>45 U/L) and good at the higher cut-off value recommended by the manufacturer (>60 U/L). The agreement is highest when two separate cut-off values are used – the lower (>55 U/L) corresponding to the upper RI of the fPLI assay (>3.5 μg/L) and the higher (>70 U/L) corresponding to the cut-off value of the fPLI assay indicating pancreatitis (>5.3 μg/L). At these cut-off values, the chance-corrected agreement given by Gwet’s AC_1_ coefficient is 0.73–0.75.

With respect to the chance-corrected agreement, these results are comparable with previous studies carried out in cats using the Roche Diagnostics DGGR-lipase assay at a cut-off value of >26 U/L [10,11,12]. The chance-corrected agreement with the fPLI assay measured using Cohen’s κ ranged from 0.60 to 0.82 depending on the cut-off value of the fPLI assay-κ = 0.60 (CI 95%: 0.50, 0.70) [10] and κ = 0.63 (CI 95%: 0.43, 0.83) [12] when the cut-off value of the fPLI assay was 3.5 μg/L, and κ = 0.68 (CI 95%: 0.59, 0.77) [10], κ = 0.70 (CI 95%: 0.59, 0.81) [11], and κ = 0.82 (CI 95%: 0.66, 0.98) [12] when the cut-off value of the fPLI assay was 5.3 μg/L. To date, the Roche Diagnostics assay is the only DGGR-lipase assay validated in cats [10,12]. To enable comparison of our results with the three studies carried out so far in cats, we calculated the AC_1_ coefficient using the results obtained in two of these studies in which individual data were available [10,12]. For the fPLI assay’s cut-off value of 3.5 μg/L, AC_1_ was 0.60 (CI 95%: 0.51, 0.70) [10] and 0.64 (CI 95%: 0.45, 0.83) [12]. Our result for the optimal cut-off value of 55 U/L was higher (AC_1_ = 0.73), however CI 95% (0.54, 0.91) markedly overlapped with CI 95% from the previous studies which precluded drawing of any definitive conclusion. For the fPLI assay’s cut-off value of 5.3 μg/L, AC_1_ was 0.68 (CI 95%: 0.59, 0.77) [10] and 0.84 (CI 95%: 0.71, 0.98) [12], and these results were very similar to the AC_1_ coefficient we obtained for the optimal cut-off value of 70 U/L (0.75; CI 95%: 0.58, 0.92).

Based on the manufacturer’s information, the DGGR-lipase assay evaluated in our study had lower repeatability (precision) (CV% from 2% to 6%) compared to the Roche Diagnostics assay (CV% from 1% to 2% [10]). However, all these values were much lower than the maximum acceptable imprecision for the measurement of lipase activity in serum [22], so we doubt it could have significantly affected the results of our study.

In dogs, both RI and the agreement with canine pancreatic lipase immunoreactivity (cPLI) assay have been investigated for the DGGR-lipase assays of at least four different manufacturers and the discrepancies between them were substantial. For the Coloripase assay by NuClin Diagnostics Inc. (Northbrook, IL, USA), which was the first DGGR-lipase assay validated in dogs, the RI was ≤120 U/L [23]. For the Roche Diagnostics assay, it was ≤108 U/L [24], for the Lipase DC FS assay by DiaSys Diagnostics (Holzheim, Germany) ≤245 U/L [25], while for the Randox Laboratories assay (Crumlin, County Antrim, UK) ≤130 U/L according to the manufacturer and ≤80 U/L according to another study [26]. Surprisingly, the highest agreement between the latter assay and cPLI was at the cut-off value of 42.15 U/L (half of the assay’s RI) (κ = 0.82; 95% CI: 0.77, 0.88 and κ = 0.75; 95% CI: 0.67, 0.82, for cPLI assay at a cut-off value of 200 and 400 μg/L, respectively) [27]. For the Roche Diagnostics assay, the highest agreement with cPLI at 200 and 400 μg/L was for the cut-off value of 108 U/L (κ = 0.79; 95% CI: 0.69, 0.90) and 216 U/L (κ = 0.80; 95% CI: 0.71, 0.90), respectively [24]. On the other hand, at a very close cut-off value of 245 U/L, the DiaSys Diagnostics assay showed much lower agreement with cPLI at 400 μg/L (κ = 0.68) [25]. These data illustrate that the cut-off values for the DGGR-lipase assay should not be transferred by default from the assay of one producer to another and the laboratories should always develop their own RI, given not only the assays but also the biochemical analyzers that they use.

In our study, the chance-corrected agreement was evaluated using the AC_1_ (γ) coefficient developed by Kilem Gwet virtually 20 years ago [18,19]. The AC_1_ and κ are based on the same general principle in which the observed chance-corrected agreement (P_o_−P_e_) is compared with the maximum possible chance-corrected agreement (1−P_e_). However, they differ in the way that the chance agreement (P_e_) is calculated; P_e_ (κ) is defined as the sum of the products of marginal distributions, while P_e_ (γ) is the half of the product of the sums of marginal distributions (Table S2). As a result, in the AC_1_ coefficient the chance agreement takes values from 0 to 0.5 which is sensible as two independent raters making a fully random choice are 50% likely to agree by chance (analogically to flipping two coins). Such a constraint does not exist for P_e_ (κ), which may take any value from 0 to 1. Consequently, a high P_e_ (κ) leads to a low chance-corrected agreement. P_e_ (κ) is known to take inadequately high values in two situations, referred to as the kappa paradoxes [28,29,30]. The first one occurs when prevalence according to both raters (tests, assays) is extreme (very high or very low) and is referred to as the prevalence paradox. The second one occurs when two tests disagree much more often in one than in another combination (i.e., T1_+_ and T2_−_> T1_−_ and T2_+_ or vice versa) and is referred to as the bias paradox. The AC_1_ coefficient has been shown to be free from these paradoxes [18,19] and is therefore superior to Cohen’s κ coefficient [31]. However, despite the existence of strong analytical evidence against using Cohen’s κ [32,33], it holds firmly while the AC_1_ coefficient has so far only been used in a few veterinary studies [34,35,36,37,38,39,40]. 

The two most significant shortcomings of our study are the small number of results under analysis and the lack of any clinical information for the cats other than the biochemical laboratory measurements. The lack of clinical data precluded drawing any conclusions regarding the potential influence of the clinical condition, diagnosis and medication on the results of the compared assays. We suppose it partly accounts for the discrepancies between the fPLI and DGGR-lipase assay’s results evident in some cats (Figure 1), however we are unable to verify this suspicion. The small sample size had the strongest impact on comparing the chance-corrected agreement with previously conducted studies as well as between the subpopulations of cats. As the subpopulation sizes were unbalanced and sometimes very low (only 15 azotemic and 10 hypertriglyceridemic cats), the confidence intervals were wide and likely to overlap even if the coefficient values were apparently distinct. Therefore, we could not perform reliable statistical comparisons and draw conclusions regarding the influence of these potential confounders on the chance-corrected agreement. In our study, the AC_1_ coefficient for the optimal cut-off of 55 U/L seemed to be lower in azotemic cats. Two studies [13,14], one published only as an abstract [14], showed a significant yet weak positive correlation between the creatinine concentration and DGGR-lipase activity. There was, however, no correlation between the DGGR-lipase activity and symmetric dimethylarginine (SDMA) concentration [13], regarded as the most sensitive indicator of an impaired glomerular filtration in cats [41]. A large-scale study carried out on 251 cats [10] did not show any influence of azotemia on the agreement between the DGGR-lipase activity and fPLI. Moreover, in our study the AC_1_ coefficient calculated for the optimal cut-off value of the DGGR-lipase assay of 70 U/L and fPLI assay at 5.3 μg/L was virtually identical in azotemic and non-azotemic cats. Therefore, we do not have any grounds to suspect that any true difference between azotemic and non-azotemic cats exists. The correlations with the creatinine concentration reported previously for the fPLI and DGGR-lipase activity were weaker than shown in our study. However, due to the lack of clinical data, we were not able to divide our cats into healthy and diseased (as was done in one of the previous studies [13]) or adjust the analysis by potential interference of other conditions (also as was done in other study [14]). Consequently, we measured the overall correlation which was very likely affected by the cats’ clinical condition. Although interference by increased concentrations of serum triglycerides was noted when the DGGR-lipase assay was being developed [8], we did not find any differences between cats with and without increased concentrations of lipid metabolites (cholesterol and triglycerides).

## 5. Conclusions

To conclude, our study confirms that the chance-corrected agreement between the PZ Cormay S.A. (Łomianki, Poland) DGGR lipase assay and the IDEXX Laboratories fPLI assay is good. Prospective studies comparing both assays to a diagnostic gold standard are needed to determine which of them is more accurate. 

## Figures and Tables

**Table 1:** Chance-corrected agreement (expressed as Gwet’s AC_1_ coefficient with the 95% confidence interval [CI 95%]) between feline pancreatic lipase immunoreactivity (fPLI) assay and DGGR-lipase assay at various cut-off values of the DGGR-lipase assay.

DGGR-Lipase Assay Cut-Off Value [U/L]	fPLI			
	>3.5 μg/L		>5.3 μg/L	
	Observed Agreement	AC_1_ (CI 95%)	Observed Agreement	AC_1_ (CI 95%)
26 ^a^	0.580	0.247 (0.002, 0.492)	0.500	0.063 (−0.196, 0.323)
45 ^b^	0.780	0.562 (0.333, 0.790)	0.700	0.400 (0.146, 0.654)
60 ^c^	0.820	0.651 (0.445, 0.858)	0.820	0.663 (0.463, 0.862)
Optimal ^d^	0.860	0.725 (0.537, 0.914)	0.860	0.749 (0.577, 0.921)

^a^ cut-off value reported in the literature for the Roche Diagnostics DGGR-lipase assay [10]; ^b^ cut-off value used by the laboratory; ^c^ cut-off value recommended by the DGGR-lipase assay’s manufacturer; ^d^ 55 U/L for fPLI assay at the cut-off value of 3.5 μg/L and 70 U/L for fPLI assay at the cut-off value of 5.3 μg/L

**Table 2:** Chance-corrected agreement (expressed as Gwet’s AC_1_ coefficient with the 95% confidence interval [CI 95%]) between feline pancreatic lipase immunoreactivity (fPLI) assay and DGGR-lipase assay at optimal cut-off values in subpopulations of cats.

Subpopulation of Cats	No. of Cats	fPLI > 3.5 μg/L and DGGR-Lipase Assay > 55 U/L		fPLI > 5.3 μg/L and DGGR-Lipase Assay > 70 U/L	
		Observed Agreement	AC_1_ (CI 95%)	Observed Agreement	AC_1_ (CI 95%)
Creatinine					
Normal (≤1.8 mg/dL)	35	0.886	0.807 (0.629, 0.985)	0.857	0.795 (0.629, 0.961)
Elevated (>1.8 mg/dL)	15	0.800	0.689 (0.373, 1.00)	0.867	0.760 (0.450, 1.00)
Cholesterol					
Normal (≤200 mg/dL)	25	0.840	0.748 (0.522, 0.974)	0.840	0.765 (0.553, 0.976)
Elevated (>200 mg/dL)	25	0.880	0.773 (0.532, 1.00)	0.880	0.762 (0.508, 1.00)
Triglycerides					
Normal (≤160 mg/dL)	40	0.850	0.703 (0.484, 0.922)	0.850	0.725 (0.522, 0.928)
Elevated (>160 mg/dL)	10	0.900	0.817 (0.475, 1.00)	0.900	0.840 (0.542, 1.00)

## Data Availability

Data is contained within the Supplementary Material Table S1.

## Supplementary Materials

The following are available online at www.mdpi.com/article/10.3390/ani11113172/s1, Table S1: Data used in the study, Table S2: Calculation of the chance-corrected agreement, Table S3: Agreement between feline pancreatic lipase immunoreactivity (fPLI) assay and DGGR lipase assay.
