# Supplementary material for: The Agreement between Feline Pancreatic Lipase Immunoreactivity and DGGR-Lipase Assay in Cats—Preliminary Results
Source: Animals (Basel). 2021 Nov 6;11(11):3172. doi: 10.3390/ani11113172 (PMC8614432; doi:10.3390/ani11113172)
Supplement: Supplementary file 1 [file animals-11-03172-s001.zip › Table S2.pdf]

Table S2. Calculation of the chance-corrected agreement

Contingency table presenting counts:

		Test 2		Totals
		+	–	
Test 1	+	a	b	$T1_+$
	–	c	d	$T1_-$
Totals		$T2_+$	$T2_-$	N

Contingency table presenting probabilities:

		Test 2		Totals
		+	–	
Test 1	+	$p_{++} = a/N$	$p_{+-} = b/N$	$p_{+.} = (a+b)/N$
	–	$p_{-+} = c/N$	$p_{--} = d/N$	$p_{-.} = (c+d)/N$
Totals		$p_{.+} = (a+c)/N$	$p_{.-} = (b+d)/N$	(a+b+c+d)/N = 100%

Calculation of the expected chance agreement (P_e) according to Cohen (1960):

$$P_e(\kappa) = \frac{T1_+ \times T2_+ + T1_- \times T2_-}{N^2} = p_{+.} \times p_{.+} + p_{-.} \times p_{.-} = (p_{+.} + p_{.+}) + (1 - p_{+.}) \times (1 - p_{.+})$$

Calculation of the expected chance agreement (P_e) according to Gwet (2002, 2008):

$$P_e(\gamma) = 2 \times \left(\frac{(T1_+ + T2_+)/2}{N}\right) \times \left(\frac{(T1_- + T2_-)/2}{N}\right) = 2 \times \frac{(p_{+.} + p_{.+})}{2} \times \frac{(p_{-.} + p_{.-})}{2}$$
$$= 2 \times \frac{(p_{+.} + p_{.+})}{2} \times \left(1 - \frac{p_{+.} + p_{.+}}{2}\right) = \frac{(p_{+.} + p_{.+}) \times (p_{-.} + p_{.-})}{2}$$
$$= \frac{(p_{+.} + p_{.+}) \times [2 - (p_{+.} + p_{.+})]}{2}$$

Calculation of the observed agreement (P_o):

$$P_o = \frac{a + d}{N} = p_{++} + p_{--}$$

Calculation of the chance-corrected (beyond chance) agreement:

$$AC_1 = \frac{P_o - P_e}{1 - P_e} = 1 - \frac{1 - P_o}{1 - P_e}$$
