# Supplementary material for: The Agreement between Feline Pancreatic Lipase Immunoreactivity and DGGR-Lipase Assay in Cats—Preliminary Results
Source: Animals (Basel). 2021 Nov 6;11(11):3172. doi: 10.3390/ani11113172 (PMC8614432; doi:10.3390/ani11113172)
Supplement: Supplementary file 1 [file animals-11-03172-s001.zip › Table S3.pdf]

Table S3. Agreement between feline pancreatic lipase immunoreactivity (fPLI) and DGGR lipase assay

Optimal cut-off values for DGGR lipase assay:

		fPLI [µg/L]		Totals
		> 3.5	≤ 3.5	
DGGR lipase assay [U/L]	> 55	18	1	19
	≤ 55	6	25	31
Totals		24	26	50

Gwet's AC_1 (γ) coefficient = 0.725 (CI 95%: 0.537, 0.914)

Cohen's kappa (κ) coefficient = 0.717 (CI 95%: 0.523, 0.912)

		fPLI [µg/L]		Totals
		> 5.3	≤ 5.3	
DGGR lipase assay [U/L]	> 70	13	0	13
	≤ 70	7	30	37
Totals		20	30	50

Gwet's AC_1 (γ) coefficient = 0.749 (CI 95%: 0.577, 0.921)

Cohen's kappa (κ) coefficient = 0.690 (CI 95%: 0.477, 0.903)

Cut-off values for DGGR lipase assay used by the veterinary laboratory:

		fPLI [µg/L]		Totals
		> 3.5	≤ 3.5	
DGGR lipase assay [U/L]	> 45	21	8	29
	≤ 45	3	18	21
Totals		24	26	50

Gwet's AC_1 (γ) coefficient = 0.562 (CI 95%: 0.333, 0.790)

Cohen's kappa (κ) coefficient = 0.563 (CI 95%: 0.335, 0.791)

		fPLI [µg/L]		Totals
		> 5.3	≤ 5.3	
DGGR lipase assay [U/L]	> 45	17	12	29
	≤ 45	3	18	21
Totals		20	30	50

Gwet's AC_1 (γ) coefficient = 0.400 (CI 95%: 0.146, 0.654)

Cohen's kappa (κ) coefficient = 0.419 (CI 95%: 0.172, 0.665)

Cut-off values for DGGR lipase assay recommended by the manufacturer:

		fPLI [µg/L]		Totals
		> 3.5	≤ 3.5	
DGGR lipase assay [U/L]	> 60	16	1	17
	≤ 60	8	25	33
Totals		24	26	50

Gwet's AC_1 (γ) coefficient = 0.651 (CI 95%: 0.445, 0.858)

Cohen's kappa (κ) coefficient = 0.635 (CI 95%: 0.420, 0.851)

		fPLI [µg/L]		Totals
		> 5.3	≤ 5.3	
DGGR lipase assay [U/L]	> 60	14	3	17
	≤ 60	6	27	33
Totals		20	30	50

Gwet's AC_1 (γ) coefficient = 0.663 (CI 95%: 0.463, 0.862)

Cohen's kappa (κ) coefficient = 0.615 (CI 95%: 0.388, 0.843)

Cut-off values for DGGR lipase assay reported in the literature:

		fPLI [µg/L]		Totals
		> 3.5	≤ 3.5	
DGGR lipase assay [U/L]	> 26	23	20	43
	≤ 26	1	6	7
Totals		24	26	50

Gwet's AC_1 (γ) coefficient = 0.247 (CI 95%: 0.002, 0.492)

Cohen's kappa (κ) coefficient = 0.184 (CI 95%: -0.008, 0.449)

		fPLI [µg/L]		Totals
		> 5.3	≤ 5.3	
DGGR lipase assay [U/L]	> 26	19	24	43
	≤ 26	1	6	7
Totals		20	30	50

Gwet's AC_1 (γ) coefficient = 0.063 (CI 95%: -0.196, 0.323)

Cohen's kappa (κ) coefficient = 0.126 (CI 95%: -0.116, 0.368)
